# Exploring the Impact of Organic Solvent Quality and Unusual Adduct Formation during LC-MS-Based Lipidomic Profiling

**DOI:** 10.3390/metabo13090966

**Published:** 2023-08-22

**Authors:** Tomas Cajka, Jiri Hricko, Lucie Rudl Kulhava, Michaela Paucova, Michaela Novakova, Oliver Fiehn, Ondrej Kuda

**Affiliations:** 1 Institute of Physiology of the Czech Academy of Sciences, Videnska 1083, 14200 Prague, Czech Republic; jiri.hricko@fgu.cas.cz (J.H.); lucie.kulhava@fgu.cas.cz (L.R.K.); michaela.paucova@fgu.cas.cz (M.P.); michaela.novakova@fgu.cas.cz (M.N.); ondrej.kuda@fgu.cas.cz (O.K.); 2 West Coast Metabolomics Center, University of California Davis, 451 Health Sciences Drive, Davis, CA 95616, USA; ofiehn@ucdavis.edu

**Keywords:** lipidomics, metabolomics, liquid chromatography, mass spectrometry, method development, solvent quality, adduct formation, MS/MS annotation, misidentification, lipids

## Abstract

Liquid chromatography–mass spectrometry (LC-MS) is the key technique for analyzing complex lipids in biological samples. Various LC-MS modes are used for lipid separation, including different stationary phases, mobile-phase solvents, and modifiers. Quality control in lipidomics analysis is crucial to ensuring the generated data’s reliability, reproducibility, and accuracy. While several quality control measures are commonly discussed, the impact of organic solvent quality during LC-MS analysis is often overlooked. Additionally, the annotation of complex lipids remains prone to biases, leading to potential misidentifications and incomplete characterization of lipid species. In this study, we investigate how LC-MS-grade isopropanol from different vendors may influence the quality of the mobile phase used in LC-MS-based untargeted lipidomic profiling of biological samples. Furthermore, we report the occurrence of an unusual, yet highly abundant, ethylamine adduct [M+46.0651]^+^ that may form for specific lipid subclasses during LC-MS analysis in positive electrospray ionization mode when acetonitrile is part of the mobile phase, potentially leading to lipid misidentification. These findings emphasize the importance of considering solvent quality in LC-MS analysis and highlight challenges in lipid annotation.

## 1. Introduction

Lipidomics, as a subsection of metabolomics, offers a comprehensive view of numerous lipids in biological samples [1]. Over the last decade, advances in chromatographic and mass spectrometric techniques, instrumental file processing, compound annotation, and bioinformatics tools for data interpretation [2,3] have led to groundbreaking discoveries, providing a better understanding of lipid metabolism, signaling, and their roles in various cellular processes and disease pathways [4,5].

Comprehensive lipidome analysis employs two main profiling approaches: liquid chromatography coupled to mass spectrometry (LC-MS) and direct infusion methods with MS analyses. LC-MS has emerged as a powerful approach, offering several advantages over direct infusion MS [6], including the ability to separate and detect isobars and isomers, reduce ion-suppression effects, and allow the separation of compounds based on their physicochemical properties [2]. Various LC-MS modes are available to separate complex lipids, including different stationary-phase chemistries, column diameters, and compositions of mobile-phase solvents and modifiers. Reversed-phase LC (RPLC) represents the most frequently used chromatography-based method for lipid separation, followed by hydrophilic interaction chromatography (HILIC), normal-phase LC (NPLC), and, more recently, supercritical fluid chromatography (SFC) [1,3].

While comparing column diameters [7], stationary-phase chemistries [8,9,10,11,12], and mobile phase modifiers [8,13,14,15], the impact of organic solvent quality during LC-MS analysis is often overlooked. As a common rule, LC-MS-grade solvents and modifiers should be used [16,17], but it is unclear how variations in solvent quality may affect LC-MS-based untargeted lipidomic analysis and data integrity.

Along with advances in LC-MS, tools for annotating complex lipids have dramatically improved using computer-generated (in silico) MS/MS databases [18], such as LipidBlast [19], LIQUID [20], or LipidHunter2 [21]. However, despite this improvement, the annotation of complex lipids remains prone to biases [22,23], leading to potential misidentifications and incomplete characterization of lipid species.

Here, we show how LC-MS-grade isopropanol from different vendors may influence the quality of the mobile phase used in LC-MS-based untargeted lipidomic profiling of biological samples. Furthermore, we report the occurrence of an unusual ethylamine adduct that may form for specific lipid subclasses during LC-MS analysis in electrospray ionization (ESI) in positive mode, potentially leading to lipid misidentification.

## 2. Materials and Methods

### 2.1. Materials and Reagents

LC-MS-grade solvents for mobile phases included acetonitrile (Honeywell, catalog no.: 34967), methanol (J.T.Baker, catalog no.: 9822), water (VWR, catalog no.: 83645.320), and isopropanol (Fisher Chemical, catalog no.: A461-212; Honeywell, catalog no.: 34965; J.T.Baker, catalog no.: 9827-02; Supelco, catalog no.: 1027812500; VWR, catalog no.: 84881.320). For sample extraction, methanol (J.T.Baker, catalog no.: 9822), methyl *tert*-butyl ether (Honeywell, catalog no.: 34875), and water (VWR, catalog no.: 83645.320) were used. Mobile phase modifiers such as ammonium formate (Supelco, catalog no.: 70221) and formic acid (VWR, catalog no.: 84865.260) were also of LC-MS-grade quality.

The internal standards used for the system suitability test (SST) were obtained from Avanti Polar Lipids/Merck and contained the following analytes: CE 22:1, Cer d18:1/17:0, cholesterol-*d*_7_, DG 12:0/12:0/0:0, DG 18:1/2:0/0:0, Hex-Cer d18:1/17:0, LPC 17:1, LPE 17:1, MG 17:0/0:0/0:0, PC 15:0/18:1-*d*_7_, PE 17:0/17:0, PG 17:0/17:0, PI 15:0/18:1-*d*_7_, PS 17:0/17:0, SM d18:1/17:0, sphingosine d17:1, TG 17:0/17:1/17:0-*d*_5_, and TG 20:0/20:1/20:0-*d*_5_ [24].

Human serum (catalog no.: S7023-100ML) and NIST SRM 1950 plasma (catalog no.: NIST1950) were obtained from Merck. The 3T3-L1 cells and mouse white adipose tissue (WAT) were from the Laboratory of Metabolism of Bioactive Lipids, and the rat liver was from the Department of Biological Control of the Institute of Physiology of the Czech Academy of Sciences [13,24].

### 2.2. Sample Preparation

Samples were extracted using protocols published before [13,24]. Specifically, 25 µL serum or plasma sample was mixed with 765 μL of ice-cold methanol/MTBE mixture (165 µL methanol and 600 µL MTBE, respectively) containing internal standards (see Ref. [24]) and this mixture was shaken (30 s). Then, 165 µL of 10% methanol was added, vortexed (10 s), and centrifuged (24,328× *g*, 5 min, 4 °C). An aliquot of 100 µL of the upper phase was collected, evaporated, and the dry serum extracts were resuspended in 100 µL methanol containing an internal standard (CUDA), shaken (30 s), centrifuged (24,328× *g*, 5 min, 4 °C), and used for LC-MS analysis [13,24].

In a 2 mL tube, the 3T3-L1 cells (as pellets), liver (20 mg), and WAT (20 mg) were homogenized (1.5 min) with 275 μL of methanol using a grinder. Then, 1 mL of MTBE was added, and this mixture was shaken (30 s). Finally, 275 μL of 10% methanol was added, and after vortexing (10 s), the tubes were centrifuged (24,328× *g*, 5 min, 4 °C) [13]. For 3T3-L1 cells, liver, and WAT an aliquot of 50 µL, 100 µL, and 10 µL the upper phase was collected, evaporated, and the dry extracts were resuspended in 100 µL, 300 µL, and 1000 µL of methanol containing an internal standard (CUDA), shaken (30 s), centrifuged (24,328× *g*, 5 min, 4 °C), and used for LC-MS analysis [13,25].

### 2.3. LC-MS Conditions

The LC-MS systems consisted of (i) a Vanquish UHPLC system (Thermo Fisher Scientific, Bremen, Germany), an Ion Max API ion source with a heated electrospray ionization (HESI-II) probe (Thermo Fisher Scientific), and a Q Exactive Plus mass spectrometer (Thermo Fisher Scientific), and (ii) a Vanquish UHPLC system (Thermo Fisher Scientific), an OptaMax NG ion source with a HESI-II probe (Thermo Fisher Scientific), and an Orbitrap Exploris 480 mass spectrometer (Thermo Fisher Scientific).

The lipids were separated on an ACQUITY Premier BEH C18 column (50 mm × 2.1 mm i.d.; 1.7 μm particle size) equipped with a VanGuard FIT cartridge (5 mm × 2.1 mm i.d.; 1.7 μm particle size) (Waters, Milford, MA, USA) [24]. The LC column was operated at a temperature of 65 °C with a flow rate of 0.6 mL/min. LC-MS analysis was performed in positive ESI mode with the following mobile phases: (A) 60:40 acetonitrile/water with 10 mM ammonium formate and 0.1% formic acid; (B) 90:10:0.1 isopropanol/acetonitrile/water with the same type of mobile phase modifiers. Separation was conducted using the following gradient: 0 min 15% (B); 0–1 min from 15% to 30% (B); 1–1.3 min from 30% to 48% (B); 1.3–5.5 min from 48% to 82% (B); 5.5–5.8 min from 82% to 99% (B); 5.8–6 min 99% (B); 6–6.1 min from 99% to 15% (B); 6.1–7 min 15% (B) +1 min preinjection steps [13]. An injection volume of 2 µL, 0.3 μL, 5 µL, and 5 µL was used for serum, cell, liver, and WAT extracts, respectively. The sample temperature was maintained at 4 °C.

The ion source and MS parameters of the Q Exactive Plus mass spectrometer were: spray voltage, +3.6 kV; sheath gas pressure, 60 arbitrary units; aux gas flow, 25 arbitrary units; sweep gas flow, 2 arbitrary units; capillary temperature, 300 °C; aux gas heater temperature, 370 °C; MS1 mass range, *m*/*z* 200–1700; MS1 resolving power, 35,000 FWHM (*m*/*z* 200); the number of data-dependent scans per cycle, 3; MS/MS resolving power, 17,500 FWHM (*m*/*z* 200); normalized collision energy, 20% [13]. The ion source and MS parameters of the Qrbitrap Exploris 480 mass spectrometer were: spray voltage, +3.6 kV; sheath gas pressure, 60 arbitrary units; aux gas flow, 25 arbitrary units; sweep gas flow, 2 arbitrary units; capillary temperature, 300 °C; aux gas heater temperature, 370 °C; MS1 mass range, *m*/*z* 200–1700; MS1 resolving power, 45,000 FWHM (*m*/*z* 200); the number of data-dependent scans per cycle, 5; MS/MS resolving power, 15,000 FWHM (*m*/*z* 200); normalized collision energy, 25%. For the profiling of triacylglycerol estolides (TG-EST), the MS1 mass range *m*/*z* 1080–1300 was used. A product ion scan was used to record MS/MS spectra at a mass resolving power of 240,000 FWHM (*m*/*z* 200) for specific experiments. 

### 2.4. Data Processing

The LC-MS instrumental files from the untargeted lipidomic profiling were processed through MS-DIAL v. 4.9.221218 software [26]. The lipids were annotated using an in silico MS/MS library in MS-DIAL and an in-house retention time—*m*/*z* library (internal standards, cholesterol, MG species). High-molecular-weight impurities in the mobile phase were annotated using accurate mass (MS1) and MS/MS spectra from the NIST20 library. Thermo Scientific Xcalibur Qual Browser software v. 4.1.50 was used for isotopic pattern simulation.

The scoring for monitoring the impact of organic solvent (isopropanol) quality was performed using the following criteria: (i) maximum signal intensity during the LC-MS analysis with the running gradient (no injection) for *m*/*z* 70–1050; (ii) maximum signal intensity during the LC-MS analysis with the running gradient (no injection) for *m*/*z* 200–1700; (iii) maximum signal intensity (total ion chromatogram, TIC) during the analysis of human serum extracts for *m*/*z* 200–1700; (iv) average signal intensity of annotated lipids in human serum extracts (Table S1). Each criterion was evaluated as “good,” “moderate,” or “unacceptable.” For parameters (i) and (ii), we assigned a value of 1 to the maximum signal intensity for a particular condition within the vendor tested and then proportionally calculated the percentage for the rest. The criterion was evaluated as “good” for 0–0.33, “moderate” for 0.34–0.66, and “unacceptable” for 0.67–1. For parameters (iii) and (iv), the same calculation was used, but the criterion was considered “good” for 0.67–1, “moderate” for 0.34–0.66, and “unacceptable” for 0–0.33. The overall rank was calculated based on the outcomes of each parameter evaluated. The evaluation did not include the cost of particular organic solvents and is provided solely for informational purposes.

## 3. Results and Discussion

### 3.1. Impact of Organic Solvent Quality during LC-MS-Based Lipidomic Profiling

Organic solvent quality during LC-MS analysis is an essential factor that can influence the accuracy, sensitivity, and reproducibility of lipid analysis. LC-MS-grade solvents should meet specific criteria, including a low mass noise level, minimal organic contamination, and minimal metal content, to fulfill the high purity requirements of LC-MS [16,17]. These solvents are manufactured using additional purification processes, stringent quality control measures, and innovative packaging techniques to minimize impurities. While several quality control measures are commonly discussed in lipidomics workflows [27,28,29,30], the impact of organic solvent quality during LC-MS analysis is often overlooked.

In RPLC-MS-based untargeted lipidomic profiling using C18 or C8-modified sorbents, a weak mobile phase typically comprises a water-acetonitrile or water-methanol mixture. Conversely, a strong mobile phase typically contains isopropanol mixed with acetonitrile or methanol [2,6]. While various typical LC-MS-grade solvents such as water, acetonitrile, and methanol are available from different vendors, the choice of isopropanol is more limited. For instance, we found over 40 different kinds of LC-MS-grade alternatives for water, acetonitrile, and methanol, but only 23 for isopropanol (Table S2). However, the choice of available solvents can be overwhelming because all are declared “LC-MS-grade,” but vendors may use different technologies to deliver the final products, resulting in differences in their quality [16,17]. Based on our previous observations, we found that isopropanol is a challenging solvent for LC-MS lipidomic profiling. For our comparison, we selected five vendors delivering LC-MS-grade isopropanol to check if the quality is sufficient for untargeted lipidomic profiling.

For this evaluation, we kept the weak mobile phase (A) as 60:40 acetonitrile/water with 10 mM ammonium formate and 0.1% formic acid for all experiments, while isopropanol from different vendors was used in the strong mobile phase (B) as 90:10:0.1 isopropanol/acetonitrile/water with 10 mM ammonium formate and 0.1% formic acid.

The sequence consisted of the following injections in positive ESI mode: 4× solvent injections to equilibrate the particular platform, 1× “no injection” analysis with the running gradient, and recording mass range *m*/*z* 70–1050 to monitor masses *m*/*z* < 200, 1× “no injection” analysis, and monitoring mass range *m*/*z* 200–1700, typically used in our LC-MS lipidomics method [13,24], 1× injection of QC lipid mix (SST, Section 2.1), and 2× injections of human serum lipid extracts. Before testing the next mobile phase with a different isopropanol, the B channel of the LC unit was purged for 6 min, and the column was rinsed for 5 min with the new mobile phase (B) to remove the residues of the previous mobile phase.

**Figure 1:**
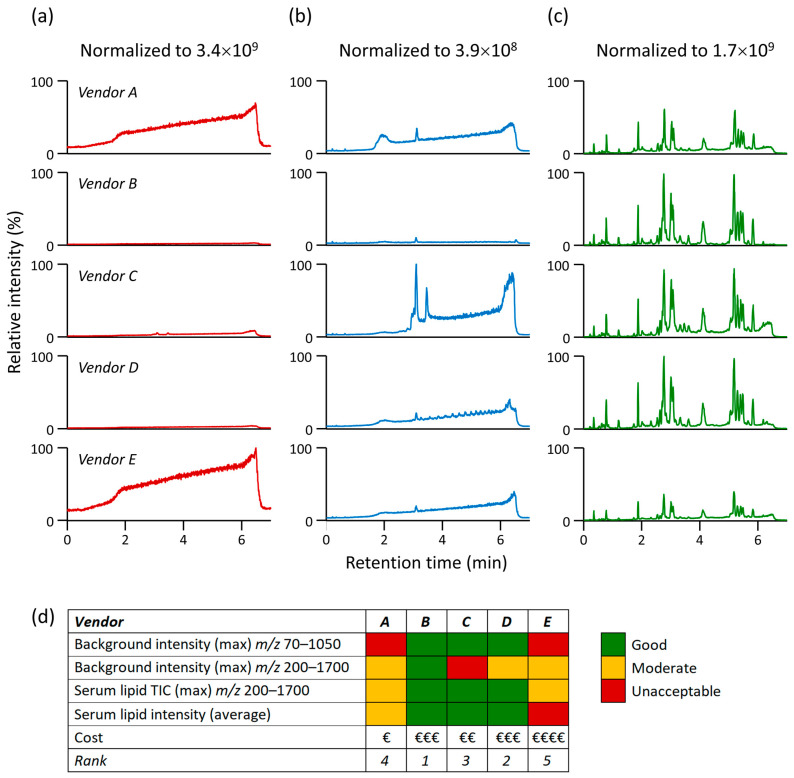
LC-MS analysis with a mobile phase containing LC-MS-grade isopropanol from five vendors in positive ESI mode. Other mobile phase components (acetonitrile, water, ammonium formate, and formic acid) were identical (see Section 2.1). Total ion chromatograms during the LC-MS analysis with the running gradient (no injection) for (**a**) the mass range *m*/*z* 70–1050, (**b**) the mass range *m*/*z* 200–1700, (**c**) human serum extracts for the mass range *m*/*z* 200–1700, and (**d**) scoring for monitoring of the impact of organic solvent (isopropanol) quality. Note: Suppliers of LC-MS-grade isopropanol are listed alphabetically in Section 2.1. However, the vendor codes (Vendor A–E) in this figure are randomized to keep them anonymized.

Figure 1a,b show significant differences observed while monitoring the background contamination from mobile phases. For the mass range *m*/*z* 70–1050, the maximum signal intensity of impurities for vendors A and E was more than 2 × 10^9^ (Figure 1a); thus, when such highly abundant ions are present, they can dominate the orbital ion trap analyzer’s capacity during the automatic gain control (AGC) process [31]. Consequently, the highly abundant ions will quickly reach the AGC target, triggering the automatic ion injection into the detector, potentially leaving insufficient capacity to adequately accumulate and detect low-abundant ions. On the other hand, mobile phases containing isopropanol from vendors B and D provided less than 4% of the maximum signal intensity, while vendor C provided 9% compared to vendor E. Of note, the high intensity of impurities can also be an issue for time-of-flight (TOF)-based analyzers because these ions hit the multichannel-plate detector, causing its faster deterioration.

After switching to the mass range *m*/*z* 200–1700, typically used in our LC-MS lipidomics method, the maximum signal intensity of impurities for vendor C was 3.9 × 10^8^, while vendors A, D, and E reached around 40% of this value, and vendor B reached only 11% (Figure 1b). This also indicates significant differences in mobile phase impurities. Although this may suggest that particular mobile phases would be suitable for LC-MS-based lipidomic profiling, the subsequent analysis of complex lipids in human serum extracts showed that for vendors A and E, the maximum signal intensity dropped to 60% and 40%, respectively, compared to vendors B, C, and D (Figure 1c).

Following the data processing of human serum lipid extracts in MS-DIAL [26] and thorough annotation of 229 lipid species spanning 15 lipid subclasses (Table S1), consistent outcomes were observed for vendors B, C, and D, with average normalized intensities ranging from 89% to 94%. In contrast, for vendors A and E, the average peak intensities dropped drastically to 42% and even as low as 24%, respectively. Figure S1 illustrates the average normalized intensities of lipid species in different lipid subclasses. The decrease in signal intensity can be explained by the fact that even though these highly abundant, low-*m*/*z* ions were not detected, they contributed to ion suppression during ESI, leading to significantly reduced signal intensities of the analyzed lipids.

**Figure 2:**
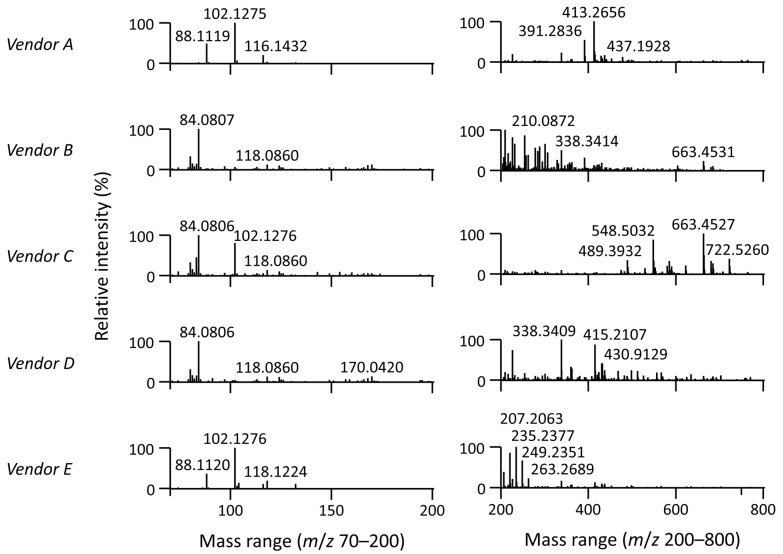
MS1 spectra of mobile phase impurities (average spectrum from 0 to 6.7 min) obtained during the LC-MS method in positive ESI mode. Each MS1 spectrum was obtained using the gradient elution (Section 2.3) with mobile phases: (A) 60:40 acetonitrile/water with 10 mM ammonium formate and 0.1% formic acid, and (B) 90:10:0.1 isopropanol/acetonitrile/water with the same type of mobile phase modifiers. LC-MS-grade isopropanol from five vendors (Section 2.1) was used to evaluate the impact of its quality during LC-MS-based lipidomic profiling. Note: Suppliers of LC-MS-grade isopropanol are listed alphabetically in Section 2.1. However, the vendor codes (Vendor A–E) in this figure are randomized to keep them anonymized.

Figure 2 shows MS1 spectra of mobile phase impurities (average spectrum from 0 to 6.7 min) obtained during LC-MS analysis in positive ESI mode. Notably, the homologous series of amines (high in isopropanol from vendors A and E) with *m*/*z* 88.112 (C_5_H_13_N, [M+H]^+^), *m*/*z* 102.128 (C_6_H_15_N, [M+H]^+^), and *m*/*z* 116.143 (C_7_H_17_N, [M+H]^+^) was detected. These amines may act as ion-pairing reagents [32], which should be avoided in LC-MS analysis. Additionally, *m*/*z* 391.284 and *m*/*z* 413.266 (high in isopropanol from vendor A) belonged to dioctyl phthalate (C_24_H_38_O_4_, [M+H]^+^ and [M+Na]^+^), used as a plasticizer [33], and *m*/*z* 338.341 (high in isopropanol from vendor D) to erucamide (C_22_H_43_NO, [M+H]^+^), used as a slip additive [34]. The *m*/*z* 663.453, annotated as tris(2,4-di-*tert*-butylphenyl)phosphate or Irgafos 168 oxide (C_42_H_63_O_4_P, [M+H]^+^) (high in isopropanol from vendor C), is an oxidized form of Irgafos 168, used as a polymer additive [35]. Moreover, *m*/*z* 548.503 was annotated as octadecyl-3-[3,5-di-*tert*-butyl-4-hydroxyphenyl]propionate] or Irganox 1076 (C_35_H_62_O_3_, [M+NH_4_]^+^), used as an antioxidant [36]. 

Figure S2 showcases the extracted ion chromatograms and MS/MS spectra of these impurities, revealing that they were frequently eluted across the entire chromatogram or exhibited broad tailing peaks. The signal intensity increased with a higher percentage of isopropanol during elution. Therefore, during the simultaneous MS1 and MS/MS acquisition, ions (*m*/*z*) associated with these peaks, as well as other background ions, should be included in the exclusion list. This precautionary step helps prevent the preferential selection of background ions over the intended lipids during precursor ion selection.

These data highlight the importance of evaluating solvent quality in LC-MS analysis, especially when developing new methods. The choice of organic solvents can significantly impact the LC-MS system’s overall performance, affecting the ionization and detection of analytes, including lipids that differ in concentration by several orders of magnitude in biological samples [22,37,38]. Moreover, manufacturing process modifications or supply chain changes can affect solvent quality without prior notice. Thus, regular monitoring and verifying purchased solvents’ quality [39] are vital to maintaining the integrity of LC-MS-based untargeted lipidomic analyses. Among the essential quality control practices, we recommend routine background noise monitoring, even for masses not usually included in the mass range of data acquisition methods. Additionally, comparing the intensity of common contaminants observed during LC-MS analysis (e.g., phthalates, Irgafos 168 oxide) over time and manufactured solvent lots, as well as regularly updating the exclusion list when simultaneous MS1 and MS/MS data acquisition is performed, should also be part of the routine practice.

### 3.2. Formation of Unusual Adducts during LC-MS-Based Lipidomic Profiling

Over the last decade, there has been a rapid increase in open-source and vendor software applications for processing mass spectral lipidomics data [40]. This is because many lipids break in an MS/MS experiment predictably; thus, fragmentation rules can be easily compiled from the literature and authentic standards for different lipid subclasses [41]. These rules are then applied to lipid structures generated using in silico methods to create a comprehensive lipidomics library for compound annotations. However, despite these improvements, the annotation of complex lipids remains prone to biases, leading to potential misidentifications [22,23,42].

Adduct formation is essential during ESI, generating different ions in both the positive and negative modes [43]. Mobile-phase modifiers (or additives) typically used at mmol/L concentrations have been shown to impact peak shape, analyte ionization efficiency, method coverage, and reproducibility of the LC-MS method, as well as the kind and intensity of formed adducts [8,13,44].

For instance, when ammonium salts (e.g., ammonium formate, ammonium acetate) are used in positive ESI mode, ammonium adducts [M+NH_4_]^+^ are observed for triacylglycerols (TG), usually accompanied by adducts with sodium and potassium ([M+Na]^+^, [M+K]^+^). However, when running a system suitability test (SST) during the LC-MS lipidomic method using a simple mixture of various lipids (see Section 2.1), we started to observe the appearance of other masses over time other than those commonly observed for these internal standards.

**Figure 3:**
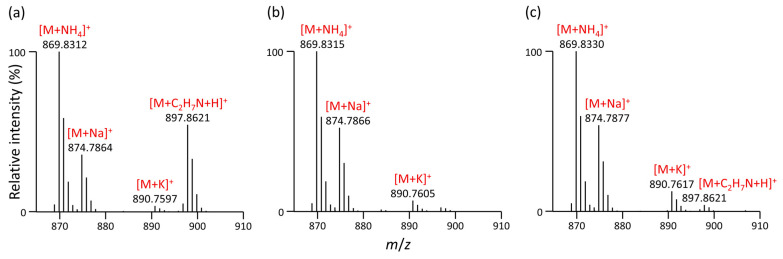
MS1 spectrum of TG 17:0/17:1/17:0-*d*_5_ acquired using RPLC separation with the following mobile phases: (**a**) containing acetonitrile and including the grounding union connecting the LC column and ESI probe; (**b**) containing methanol instead of acetonitrile and including the grounding union connecting the LC column and ESI probe, and (**c**) containing acetonitrile without the grounding union connecting the LC column and ESI probe.

Figure 3a shows the example of the MS1 spectrum of TG 51:1-*d*_5_ (TG 17:0/17:1/17:0-*d*_5_), in which case the difference between the accurate masses *m*/*z* 869.8312 [M+NH_4_]^+^ and *m*/*z* 897.8621 was 28.0309 units, indicating an alkane series of molecules differing by C_2_H_4_ (exact mass 28.0308 *m*/*z* units). However, no standard that would correspond to TG 53:1-*d*_5_ was present in this mixture. Additionally, the retention times of both masses were found to be identical, indicating a false positive. In RPLC, the lipid species are separated based on the number of double bonds and the length of fatty acyl chains (carbon number). A higher carbon number in the fatty acyl chains corresponds to increased retention times, while an additional double bond has the opposite effect and leads to faster elution [22].

This observation has raised suspicion that, in addition to the common [M+NH_4_]^+^, [M+Na]^+^, and [M+K]^+^ adducts, another adduct of TG 51:1-*d*_5_ can be formed. Based on the elemental composition calculated, we suggested that *m*/*z* 897.8621 corresponds to [M+C_2_H_8_N]^+^. In 2006, Gu et al. reported that some compounds readily form the ethylamine adduct [M+CH_3_CH_2_NH_2_+H]^+^ during ESI in positive mode, and this adduct was observed only when the mobile phase contained acetonitrile but not methanol. Ethylamine involved in the adduct was proposed to be a reduction product of acetonitrile, and the reductive hydrogen originated from water via electrolysis [45]. The occurrence of this adduct has been occasionally reported for acetic derivatives of sugars and sugar alcohols [46], triacylglycerols [47], triacylglycerol estolides [48], and phenolic glycosides [49].

After replacing acetonitrile in both mobile phases used during LC-MS lipidomic profiling with methanol, we observed that the [M+C_2_H_8_N]^+^ adduct completely disappeared (Figure 3b), confirming previous observations by Gu et al. [45]. A thorough investigation involving the replacement of stainless-steel capillaries, a column heater, an ESI needle insert, testing of PEEK capillary tubing, and experimentation with varying column temperatures (45 °C vs. 65 °C) showed that this adduct became negligible upon removal of the grounding union connecting the tubing from the LC column and ESI probe (Figure 3c), as compared to the previous setup.

Formation of ethylamine adducts [M+C_2_H_8_N]^+^ was also observed for another TG in this mixture (TG 20:0/20:1/20:0-*d*_5_) and other lipid subclasses usually forming [M+NH_4_]^+^ adducts in positive ESI mode, such as diacylglycerols (DG 12:0/12:0/0:0, DG 18:1/2:0/0:0), phosphatidylglycerols (PG 17:0/17:0), phosphatidylinositols (PI 15:0/18:1-*d*_7_), and cholesteryl esters (CE 22:1).

**Figure 4:**
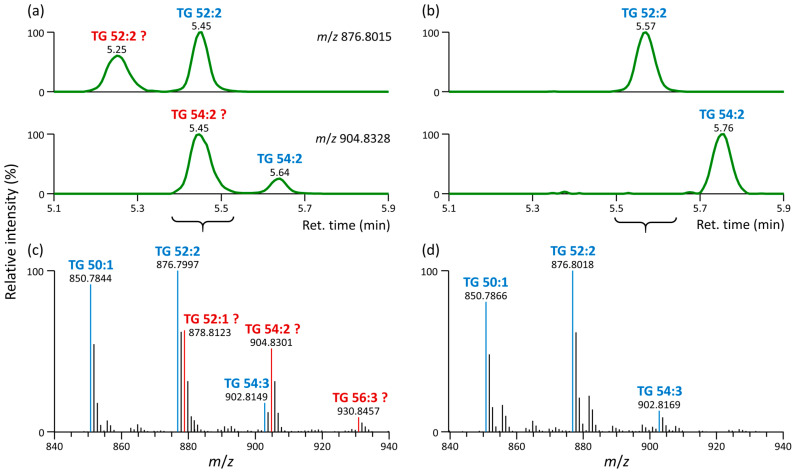
LC-MS lipidomic profiling of 3T3-L1 cell extracts. (**a**,**b**) Extracted ion chromatograms corresponding to TG 52:2 (*m*/*z* 876.8015) and TG 54:2 (*m*/*z* 904.8328), indicating true lipids (in blue) and misidentified lipids (in red) based on MS1 accurate mass only; (**c**,**d**) MS1 spectrum with annotated lipids based on MS1 accurate mass only, indicating true lipids (in blue) and misidentified lipids (in red). (**a**,**c**) Data acquired with mobile phases containing acetonitrile and including the grounding union connecting the LC column and ESI probe; (**b**,**d**) data acquired with mobile phases containing methanol instead of acetonitrile and including the grounding union connecting the LC column and ESI probe. The mobile phases were (A) 60:40 acetonitrile or methanol/water with 10 mM ammonium formate and 0.1% formic acid and (B) 90:10:0.1 isopropanol/acetonitrile or methanol/water with the same type of mobile phase modifiers.

Since the mass difference (28.0308 *m*/*z* units) between [M+NH_4_]^+^ and [M+C_2_H_8_N]^+^ adducts indicated an alkane series of molecules differing by C_2_H_4_, we were wondering if this also impacts lipid annotation. Figure 4a shows that for each TG 52:2 and TG 54:2, two theoretical species could be annotated in cell lipid extracts based on MS1 accurate mass, assuming these are ammonium adducts. In contrast, only a single species was detected and annotated when the mobile phases used methanol instead of acetonitrile, eliminating the [M+C_2_H_8_N]^+^ adduct (Figure 4b). Figure 4c,d also shows how the MS1 spectra were simplified when using methanol in the mobile phases instead of acetonitrile after eliminating ethylamine adducts. A similar observation was also noted regarding TG species in various biological matrices: human serum (Figure S3), plasma (Figure S4), rat liver (Figure S5), and mouse WAT (Figure S6). Additionally, the presence of [M+C_2_H_8_N]^+^ adducts was also observed for DG, PI, CE, and TG-EST lipid subclasses across distinct biological sources, as exemplified for rat liver (Figures S7 and S8), human plasma (Figure S9), and mouse WAT (Figure S10).

Ethylamine adducts are generally misleading, as they are structural isomers of [M+NH_4_]^+^ adducts of other TG species. For instance, TG 54:2 [M+NH_4_]^+^ and TG 52:2 [M+C_2_H_8_N]^+^ (misidentified as TG 54:2 [M+NH_4_]^+^) have the same accurate mass *m*/*z* 904.8328 but different retention times (5.64 and 5.45 min, respectively, Figure 4a). Interestingly, when checking MS/MS spectra for both TG standards (TG 17:0/17:1/17:0-*d*_5_ and TG 20:0/20:1/20:0-*d*_5_) acquired at a normalized collision energy of 20%, no informative tandem MS/MS spectra were generated from TG with ethylamine adducts, indicating that these adducts are not sufficiently stable.

However, when analyzing lipid extracts, acquired MS/MS spectra contained fragments that matched in silico MS/MS spectra (Figures S11–S14). These misleading fragments originated from ^13^C precursor ions [M+2+NH_4_]^+^ of adjacent lipids; thus, their accurate mass is slightly shifted compared to fragments from ^12^C precursor ions.

**Figure 5:**
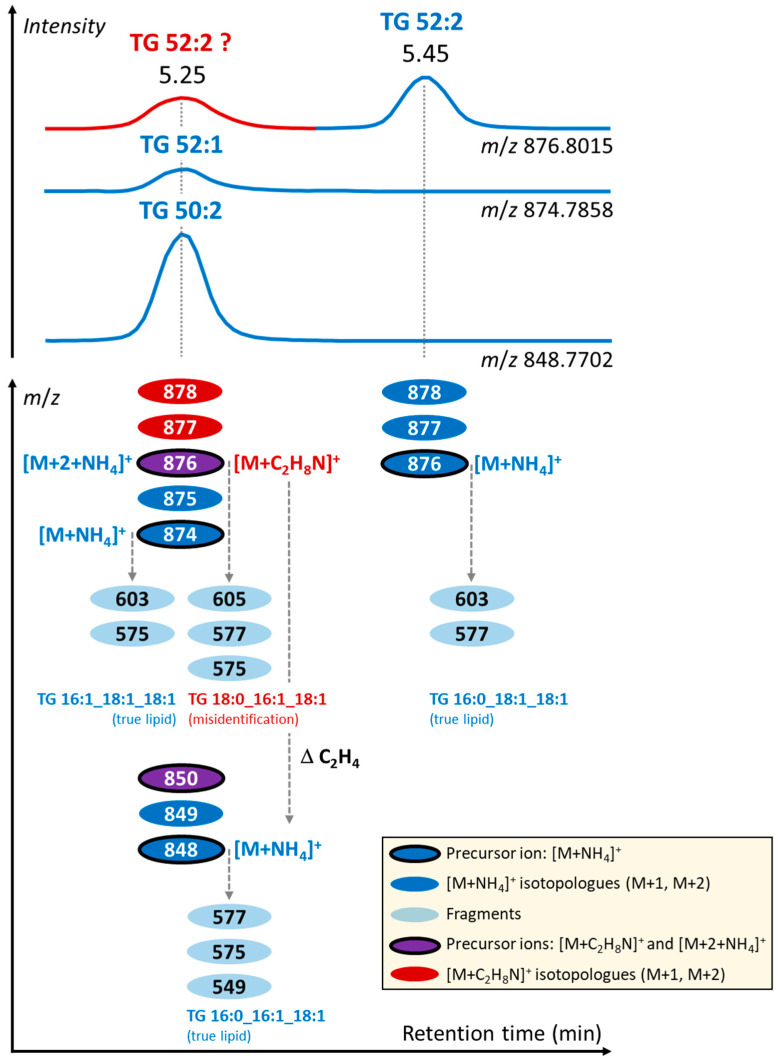
Example of annotation of TG with [M+NH_4_]^+^ and [M+C_2_H_8_N]^+^ adducts based on retention time, MS1, and MS/MS spectra.

For instance, for true TG 52:2 (TG 16:0_18:1_18:1) [M+NH_4_]^+^ with *m*/*z* 876.7997 at 5.45 min (Figure 4a, upper chromatogram), the fragments *m*/*z* 577.5178 and *m*/*z* 603.5331 were observed (Figure S11a). On the other hand, for misidentified TG 52:2 (TG 18:0_16:1_18:1) with *m*/*z* 876.7979 at 5.25 min (Figure 4a, upper chromatogram), fragments *m*/*z* 575.5074, *m*/*z* 577.5087, *m*/*z* 605.5399, and others were observed (Figure S11b), originating from the [M+2+NH_4_]^+^ ion of the adjacent lipid TG 52:3 (TG 16:1_18:1_18:1, *m*/*z* 874.7835, [M+NH_4_]^+^) with typically detected fragments *m*/*z* 575.5022 and *m*/*z* 603.5330 (Figure S11c). This misidentified TG 52:2 represents, in fact, the [M+C_2_H_8_N]^+^ adduct of TG 50:2. Figure 5 summarizes this observation for these TG species.

Similarly, for true TG 54:2 (TG 18:0_18:1_18:1) [M+NH_4_]^+^ with *m*/*z* 904.8323 at 5.64 min (Figure 4a, bottom chromatogram), the fragments *m*/*z* 603.5335 and *m*/*z* 605.5492 were observed (Figure S12a). On the other hand, for misidentified TG 54:2 (TG 18:0_18:1_18:1) with *m*/*z* 904.8328 at 5.45 min (Figure 4a, bottom chromatogram), fragments *m*/*z* 603.5320 and *m*/*z* 605.5399 were observed (Figure S12b), originating from the [M+2+NH_4_]^+^ ion of the adjacent lipid TG 54:3 (TG 18:1_18:1_18:1, *m*/*z* 902.8171, [M+NH_4_]^+^) with typically detected fragment *m*/*z* 603.5334 (Figure S12c). This misidentified TG 54:2 represented the [M+C_2_H_8_N]^+^ adduct of TG 52:2. Figures S13 and S14 provide additional examples of misidentifications in TG species.

Using a mass resolving power of 240,000 FWHM for reliable differentiation of ^12^C and ^13^C contributions to MS/MS fragments, we found that, for instance, for TG 52:2, less than 15% of the original ^12^C fragment *m*/*z* 603.5347 (from adjacent lipid TG 52:3) passed to the detector when the precursor ion was isolated with a 1 Da window, thus having a negligible impact on the mass accuracy of the dominating ^13^C *m*/*z* 603.5256 fragment from the [M+2+NH_4_]^+^ precursor ion.

Overall, when using, for instance, a 0.01 Da mass tolerance for fragment match during data processing, they can easily pass through this criterion, leading to lipid misidentification during automated lipid annotation. Therefore, manual inspection of annotated lipids based on MS/MS and considering chromatographic behavior is helpful to refine the annotation of lipid structures.

## 4. Conclusions

This study focused on the impact of organic solvent quality during LC-MS-based untargeted lipidomic profiling. Comparing LC-MS-grade isopropanol from different vendors, we identified some with high signal intensities of impurities, leading to ion suppression during ESI and affecting the intensity of complex lipids in biological samples. We further annotated the most abundant impurities to be the homologous series of amines, dioctyl phthalate, erucamide, tris(2,4-di-*tert*-butylphenyl)phosphate, and octadecyl-3-[3,5-di-*tert*-butyl-4-hydroxyphenyl]propionate. Based on these findings, we recommend routine background noise monitoring, comparing common contaminants’ intensities over time and manufactured solvent lots, and regularly updating the exclusion list when MS/MS data acquisition is used.

Next, we investigated the formation of unusual adducts during LC-MS-based lipidomic profiling and its impact on lipid annotation. We noticed signals (masses) with high abundance for certain lipid subclasses over time and in the routinely monitored masses and adducts by conducting a system suitability test. Subsequent analysis revealed the formation of an ethylamine adduct [M+C_2_H_8_N]^+^ during LC-MS analysis in positive ESI mode when the mobile phase contained acetonitrile and the grounding union connecting the LC column and ESI probe was used. We found that ethylamine adducts [M+C_2_H_8_N]^+^ can be misleading due to their similarity to [M+NH_4_]^+^ adducts typically formed for TG, DG, PI, CE, and TG-EST species. As a result, we recommend employing manual inspection of annotated lipids based on MS/MS data and considering chromatographic behavior to refine lipid annotations.

## Data Availability

Data are contained within this article and Supplementary Materials.

## Supplementary Materials

The following supporting information can be downloaded at: https://www.mdpi.com/article/10.3390/metabo13090966/s1, Figure S1: Average normalized intensity (%) of lipids per subclass from LC-MS-based untargeted analysis of human serum lipid extracts; Figure S2: Extracted ion chromatograms and MS/MS spectra of mobile phase impurities obtained during LC-MS analysis in positive ESI mode; Figure S3: LC-MS lipidomic profiling of TG species in human serum extracts; Figure S4: LC-MS lipidomic profiling of TG species in human plasma extracts (NIST SRM 1950); Figure S5: LC-MS lipidomic profiling of TG species in rat liver extracts; Figure S6: LC-MS lipidomic profiling of TG species in mouse WAT extracts; Figure S7: LC-MS lipidomic profiling of DG species in rat liver extracts; Figure S8: LC-MS lipidomic profiling of PI species in rat liver extracts; Figure S9: LC-MS lipidomic profiling of CE species in human plasma extracts (NIST SRM 1950); Figure S10: LC-MS lipidomic profiling of TG-EST species in mouse WAT extracts; Figure S11: Examples of MS/MS spectra of true and misidentified TG species in 3T3-L1 cell extracts; Figure S12: Examples of MS/MS spectra of true and misidentified TG species in 3T3-L1 cell extracts; Figure S13: Examples of MS/MS spectra of true and misidentified TG species in human serum extracts; Figure S14: Examples of MS/MS spectra of true and misidentified TG species in mouse WAT extracts; Table S1: Overview of annotated lipids in human serum extracts; Table S2: Overview of LC-MS-grade solvents (isopropanol, methanol, acetonitrile, and water).
