# Supplementary material for: Exploring the Impact of Organic Solvent Quality and Unusual Adduct Formation during LC-MS-Based Lipidomic Profiling
Source: Metabolites. 2023 Aug 22;13(9):966. doi: 10.3390/metabo13090966 (PMC10536874; doi:10.3390/metabo13090966)

metabolites

MDPI

Supplementary Materials

Exploring the Impact of Organic Solvent Quality and Unusual Adduct Formation during LC-MS-based Lipidomic Profiling

Tomas Cajka [1,*], Jiri Hricko [1], Lucie Rudl Kulhava [1], Michaela Paucova [1], Michaela Novakova [1], Oliver Fiehn [2], and Ondrej Kuda [1]

[1] Institute of Physiology of the Czech Academy of Sciences, Videnska 1083, 14200 Prague, Czech Republic

[2] West Coast Metabolomics Center, University of California, Davis, 451 Health Sciences Drive, Davis, CA, 95616, United States

* Correspondence: tomas.cajka@fgu.cas.cz

Figure S1. Average normalized intensity (%) of lipids per subclass from LC-MS-based untargeted analysis of human serum lipid extracts

Figure S2. Extracted ion chromatograms (left panels) and MS/MS spectra (right panels) of mobile phase impurities obtained during LC-MS analysis in positive ESI mode

Figure S3. LC-MS lipidomic profiling of TG species in human serum extracts

Figure S4. LC-MS lipidomic profiling of TG species in human plasma extracts (NIST SRM 1950)

Figure S5. LC-MS lipidomic profiling of TG species in rat liver extracts

Figure S6. LC-MS lipidomic profiling of TG species in mouse WAT extracts

Figure S7. LC-MS lipidomic profiling of DG species in rat liver extracts

Figure S8. LC-MS lipidomic profiling of PI species in rat liver extracts

Figure S9. LC-MS lipidomic profiling of CE species in human plasma extracts (NIST SRM 1950)

Figure S10. LC-MS lipidomic profiling of TG-EST species in mouse WAT extracts

Figure S11. Examples of MS/MS spectra of true and misidentified TG species in 3T3-L1 cell extracts

Figure S12. Examples of MS/MS spectra of true and misidentified TG species in 3T3-L1 cell extracts

Figure S13. Examples of MS/MS spectra of true and misidentified TG species in human serum extracts

Figure S14. Examples of MS/MS spectra of true and misidentified TG species in mouse WAT extracts

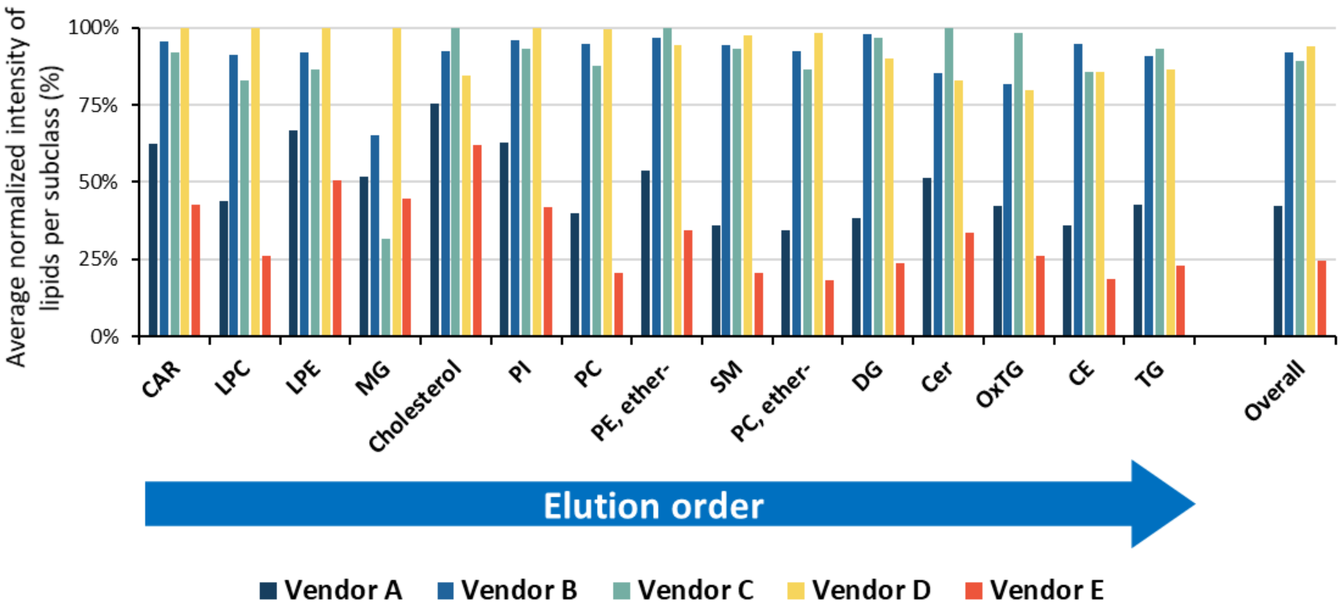

Figure S1. Average normalized intensity (%) of lipids per subclass from LC-MS-based untargeted analysis of human serum lipid extracts. For LC-MS analysis in positive ESI mode, a mobile phase (B) containing LC-MS-grade isopropanol from five vendors (A–E) was used, while other mobile phase components (acetonitrile, water, ammonium formate, formic acid) were identical (see Section 2.1). Lipid subclasses are sorted according to the median retention times of all annotated lipids per particular lipid subclass. Raw data are available in Table S1.

Amine ($C_5H_{13}N$), $[M+H]^+$, *m/z* 88.112

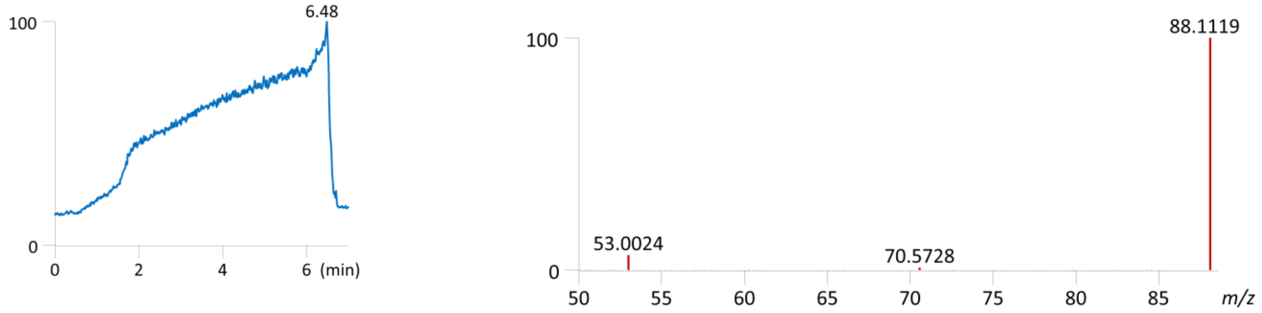

Amine ($C_6H_{15}N$), $[M+H]^+$, *m/z* 102.128

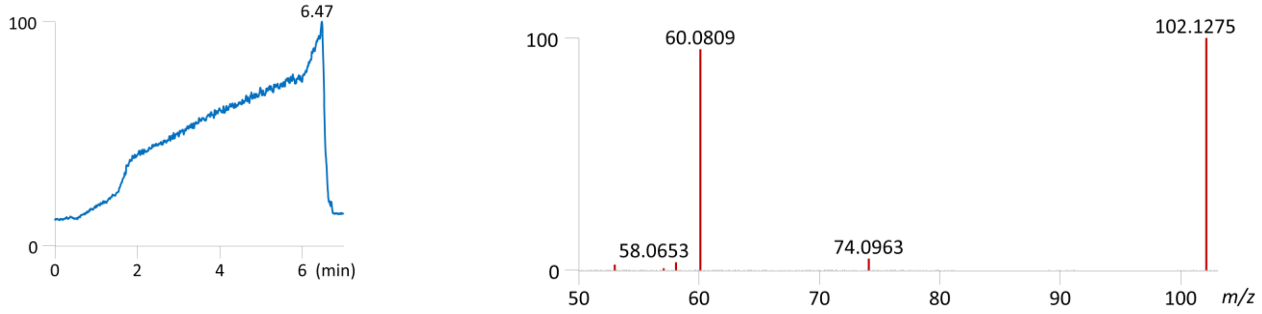

Amine ($C_7H_{17}N$), $[M+H]^+$, *m/z* 116.143

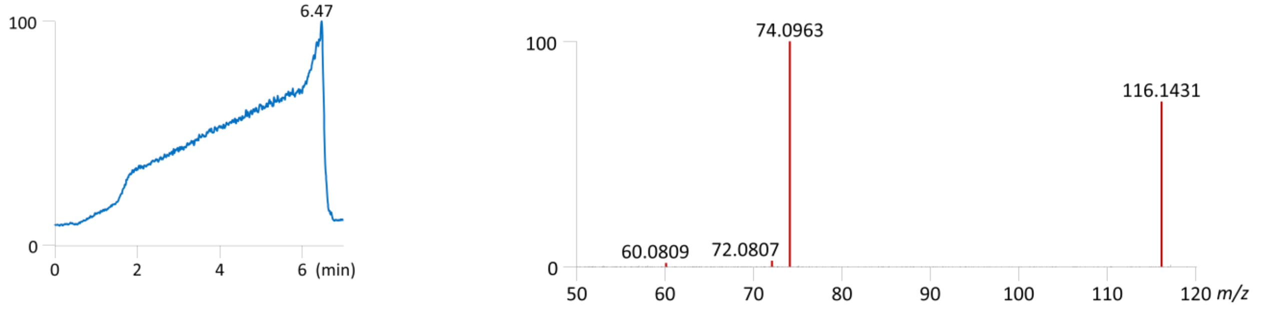

Dioctyl phthalate ($C_{24}H_{38}O_4$), $[M+H]^+$, *m/z* 391.284

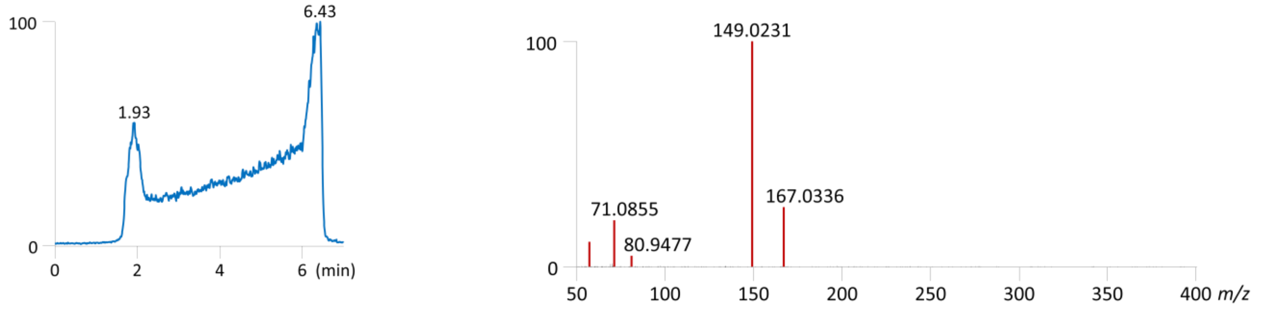

Dioctyl phthalate ($C_{24}H_{38}O_4$), $[M+Na]^+$, *m/z* 413.266

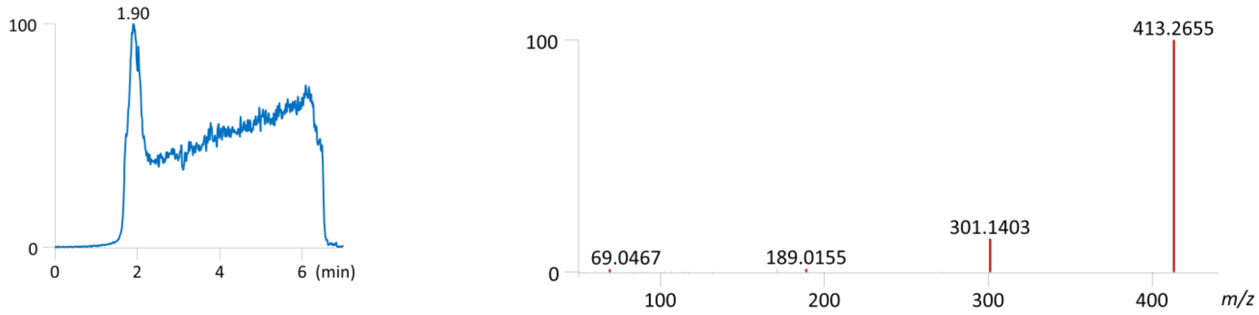

Erucamide ($C_{22}H_{43}NO$), $[M+H]^+$, *m/z* 338.341

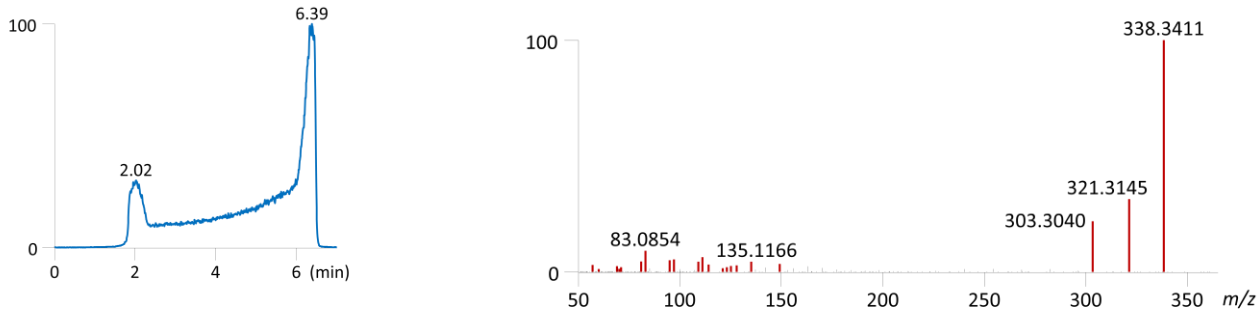

Irgafos 168 oxide ($C_{42}H_{63}O_4P$), $[M+H]^+$, *m/z* 663.453

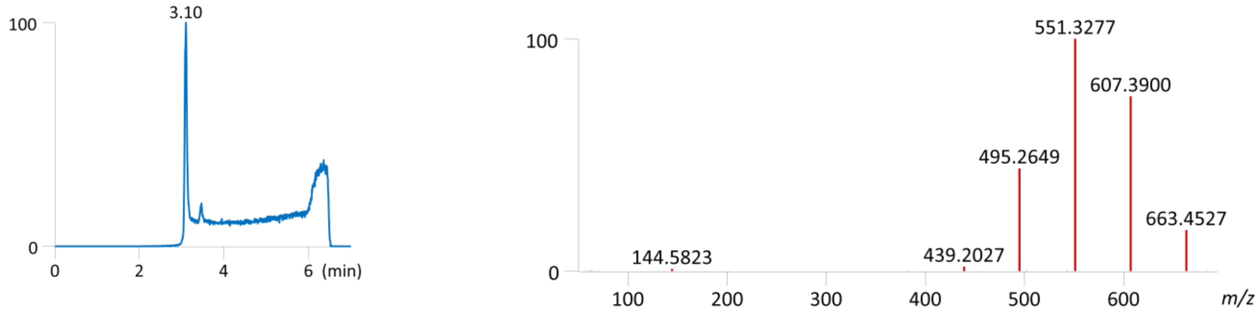

Irganox 1076 ($C_{35}H_{62}O_3$), $[M+NH_4]^+$, *m/z* 548.503

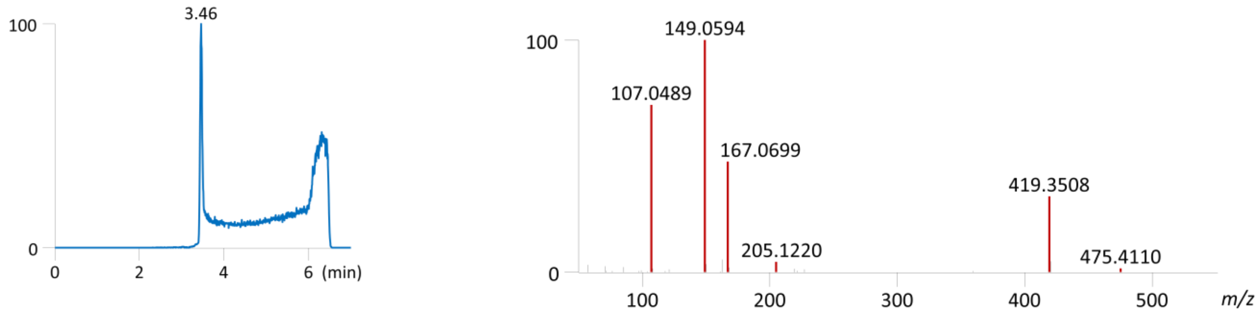

Figure S2. Extracted ion chromatograms (left panels) and MS/MS spectra (right panels) of mobile phase impurities obtained during LC-MS analysis in positive ESI mode.

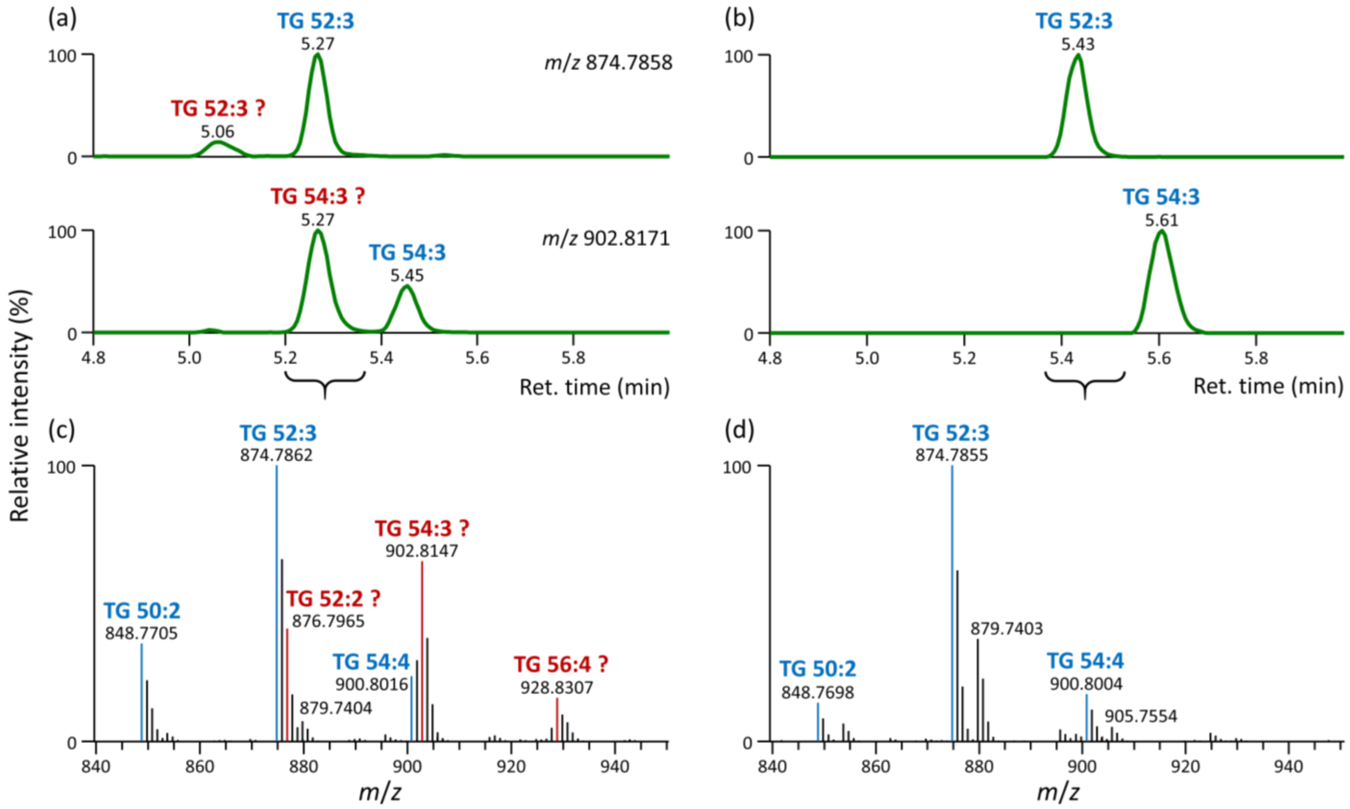

Figure S3. LC-MS lipidomic profiling of TG species in human serum extracts. (**a**,**b**) Extracted ion chromatograms corresponding to TG 52:3 (*m*/*z* 874.7858) and TG 54:3 (*m*/*z* 902.8171), indicating true lipids (in blue) and misidentified lipids (in red) based on MS1 accurate mass only; (**c**,**d**) MS1 spectrum with annotated lipids based on MS1 accurate mass only, indicating true lipids (in blue) and misidentified lipids (in red). (**a**,**c**) Data acquired with mobile phases containing acetonitrile and including the grounding union connecting the LC column and ESI probe; (**b**,**d**) data acquired with mobile phases containing methanol instead of acetonitrile and including the grounding union connecting the LC column and ESI probe. The mobile phases were (A) 60:40 acetonitrile or methanol/water with 10 mM ammonium formate and 0.1% formic acid and (B) 90:10:0.1 isopropanol/acetonitrile or methanol/water with the same type of mobile phase modifiers.

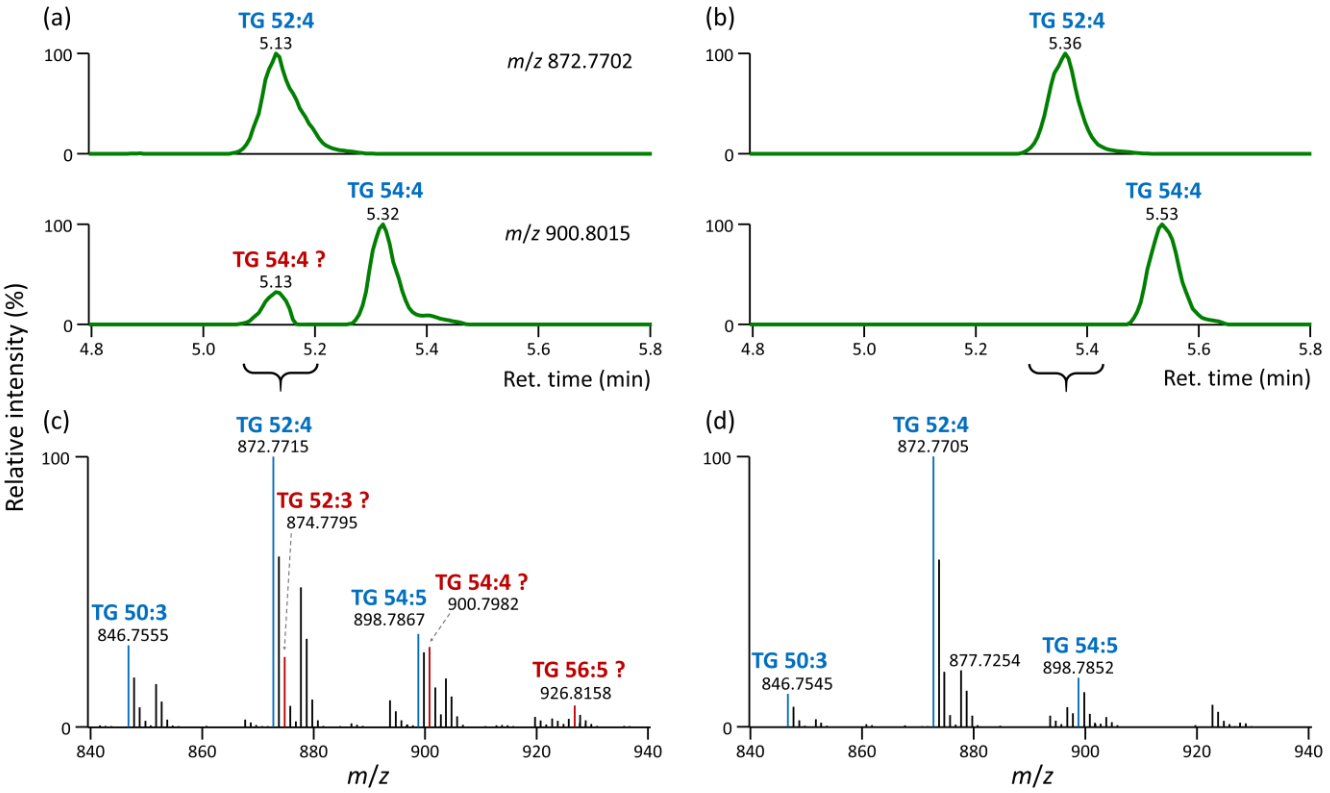

Figure S4. LC-MS lipidomic profiling of TG species in human plasma extracts (NIST SRM 1950). (**a**,**b**) Extracted ion chromatograms corresponding to TG 52:4 (*m/z* 872.7702) and TG 54:4 (*m/z* 900.8015), indicating true lipids (in blue) and misidentified lipids (in red) based on MS1 accurate mass only; (**c**,**d**) MS1 spectrum with annotated lipids based on MS1 accurate mass only, indicating true lipids (in blue) and misidentified lipids (in red). (**a**,**c**) Data acquired with mobile phases containing acetonitrile and including the grounding union connecting the LC column and ESI probe; (**b**,**d**) data acquired with mobile phases containing methanol instead of acetonitrile and including the grounding union connecting the LC column and ESI probe. The mobile phases were (A) 60:40 acetonitrile or methanol/water with 10 mM ammonium formate and 0.1% formic acid and (B) 90:10:0.1 isopropanol/acetonitrile or methanol/water with the same type of mobile phase modifiers.

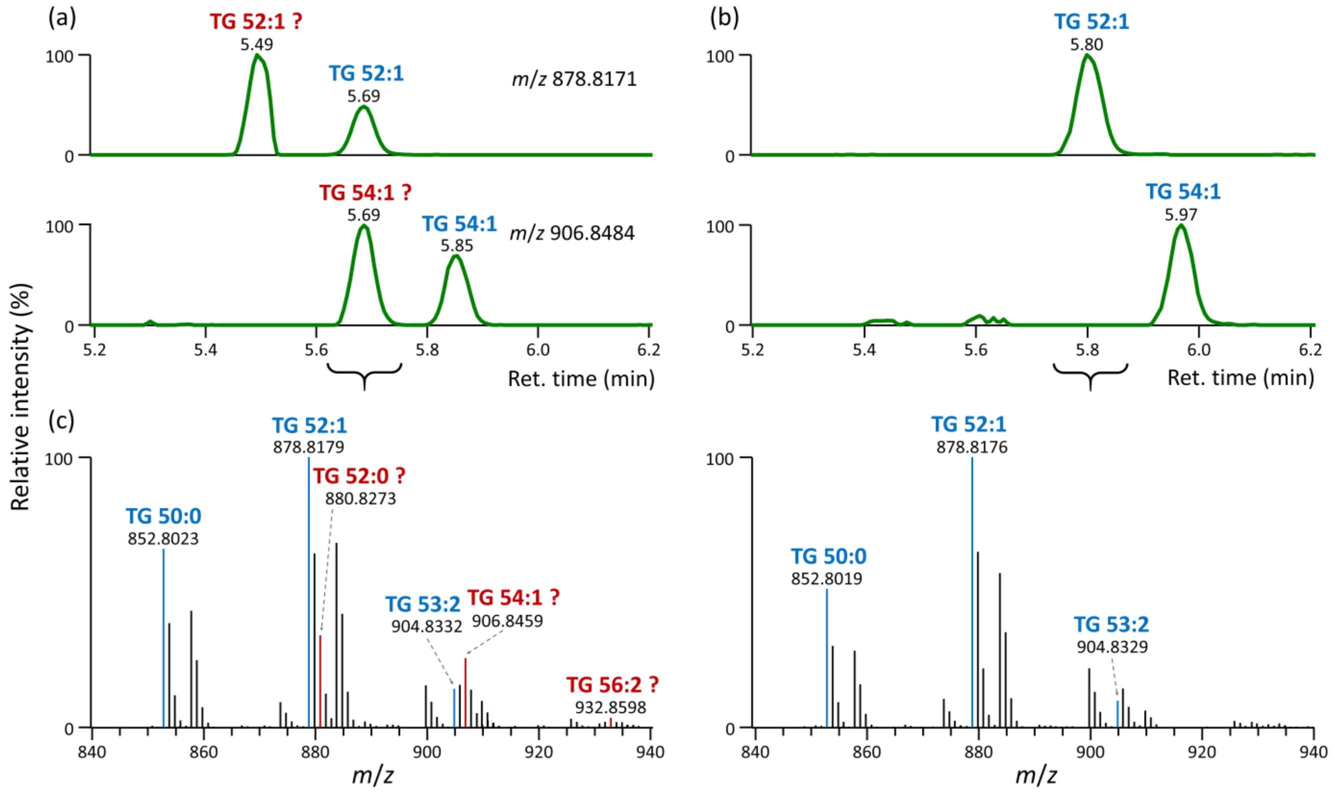

Figure S5. LC-MS lipidomic profiling of TG species in rat liver extracts. (**a**,**b**) Extracted ion chromatograms corresponding to TG 52:1 (*m*/*z* 878.8171) and TG 54:1 (*m*/*z* 906.8484), indicating true lipids (in blue) and misidentified lipids (in red) based on MS1 accurate mass only; (**c**,**d**) MS1 spectrum with annotated lipids based on MS1 accurate mass only, indicating true lipids (in blue) and misidentified lipids (in red). (**a**,**c**) Data acquired with mobile phases containing acetonitrile and including the grounding union connecting the LC column and ESI probe; (**b**,**d**) data acquired with mobile phases containing methanol instead of acetonitrile and including the grounding union connecting the LC column and ESI probe. The mobile phases were (A) 60:40 acetonitrile or methanol/water with 10 mM ammonium formate and 0.1% formic acid and (B) 90:10:0.1 isopropanol/acetonitrile or methanol/water with the same type of mobile phase modifiers.

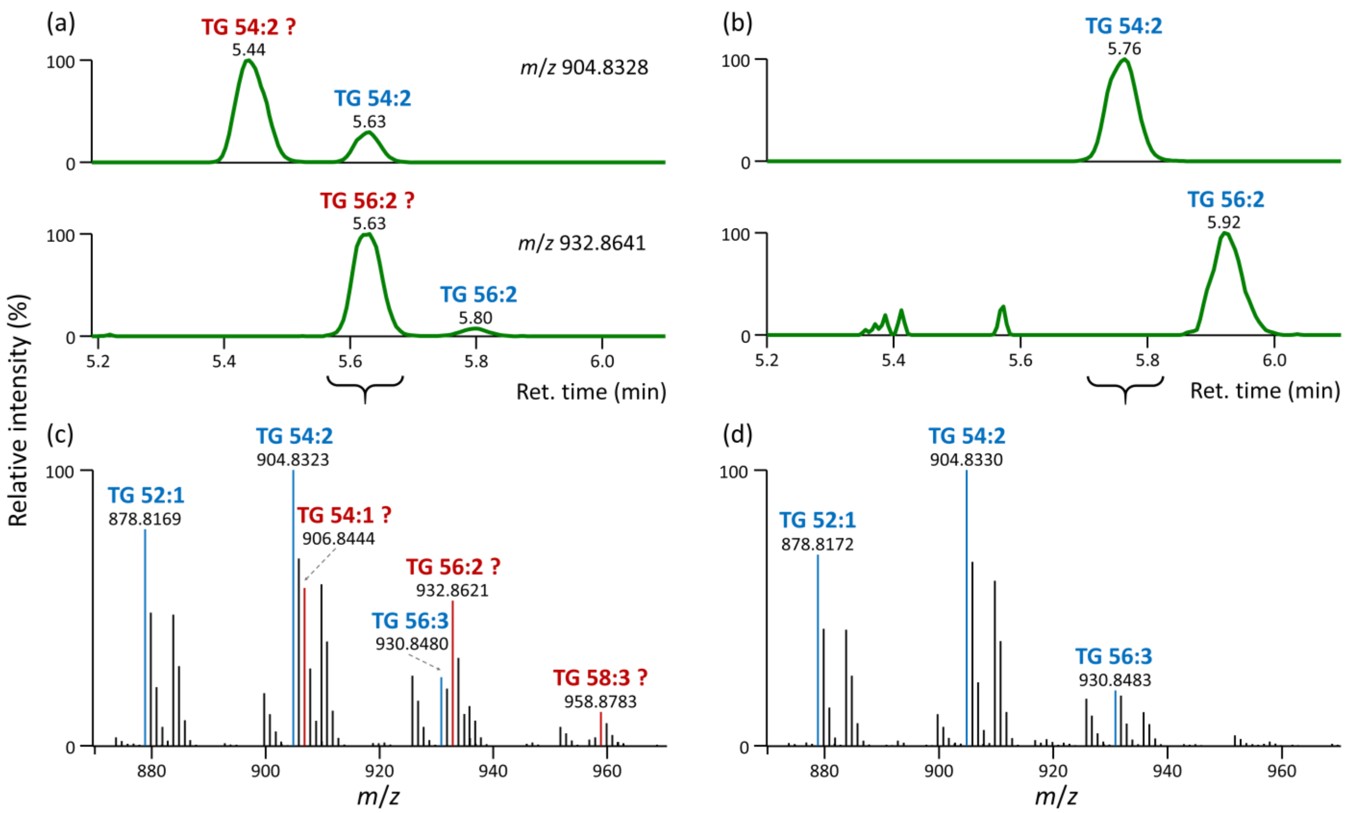

Figure S6. LC-MS lipidomic profiling of TG species in mouse WAT extracts. (**a**,**b**) Extracted ion chromatograms corresponding to TG 54:2 (*m*/*z* 904.8328) and TG 56:2 (*m*/*z* 932.8641), indicating true lipids (in blue) and misidentified lipids (in red) based on MS1 accurate mass only; (**c**,**d**) MS1 spectrum with annotated lipids based on MS1 accurate mass only, indicating true lipids (in blue) and misidentified lipids (in red). (**a**,**c**) Data acquired with mobile phases containing acetonitrile and including the grounding union connecting the LC column and ESI probe; (**b**,**d**) data acquired with mobile phases containing methanol instead of acetonitrile and including the grounding union connecting the LC column and ESI probe. The mobile phases were (A) 60:40 acetonitrile or methanol/water with 10 mM ammonium formate and 0.1% formic acid and (B) 90:10:0.1 isopropanol/acetonitrile or methanol/water with the same type of mobile phase modifiers.

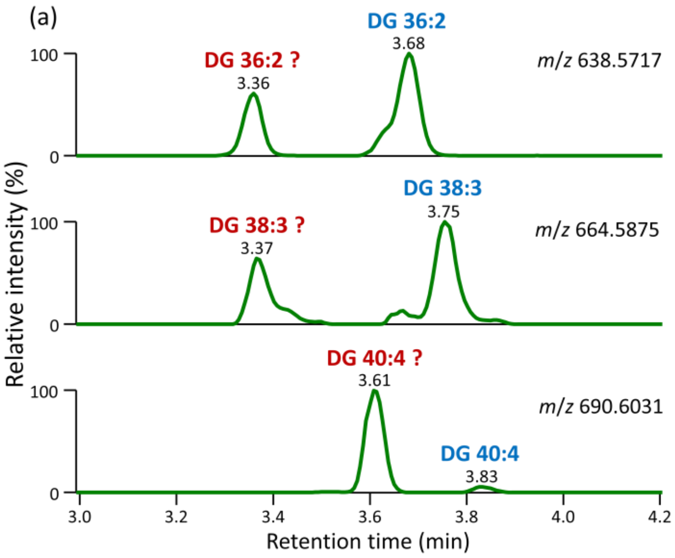

Figure S7. LC-MS lipidomic profiling of DG species in rat liver extracts. (**a**,**b**) Extracted ion chromatograms corresponding to DG 36:2 (*m*/*z* 638.5717), DG 38:3 (*m*/*z* 664.5875), and DG 40:4 (*m*/*z* 690.6031), indicating true lipids (in blue) and misidentified lipids (in red) based on MS1 accurate mass only. (**a**) Data acquired with mobile phases containing acetonitrile and including the grounding union connecting the LC column and ESI probe; (**b**) data acquired with mobile phases containing methanol instead of acetonitrile and including the grounding union connecting the LC column and ESI probe. The mobile phases were (A) 60:40 acetonitrile or methanol/water with 10 mM ammonium formate and 0.1% formic acid and (B) 90:10:0.1 isopropanol/acetonitrile or methanol/water with the same type of mobile phase modifiers.

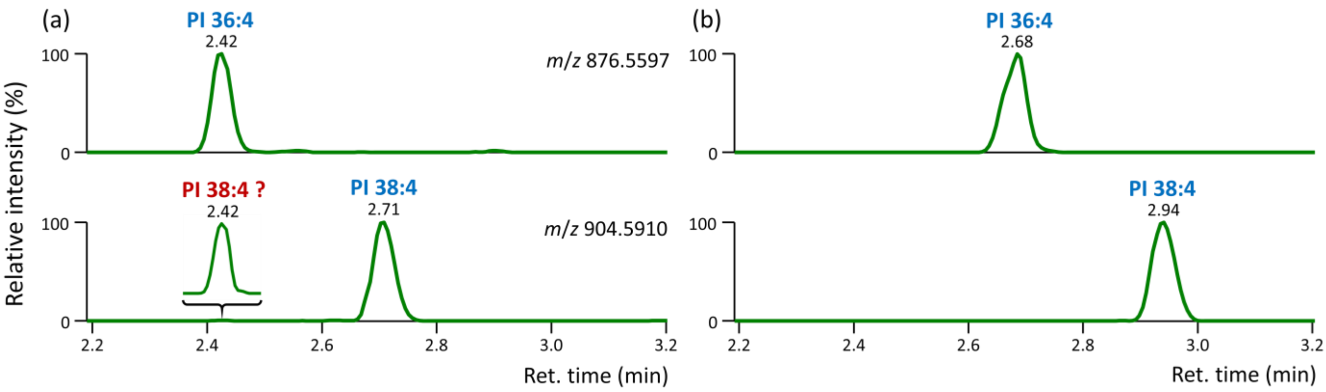

Figure S8. LC-MS lipidomic profiling of PI species in rat liver extracts. (**a**,**b**) Extracted ion chromatograms corresponding to PI 36:4 (*m*/*z* 876.5597) and PI 38:4 (*m*/*z* 904.5910), indicating true lipids (in blue) and misidentified lipids (in red) based on MS1 accurate mass only. (**a**) Data acquired with mobile phases containing acetonitrile and including the grounding union connecting the LC column and ESI probe; (**b**) data acquired with mobile phases containing methanol instead of acetonitrile and including the grounding union connecting the LC column and ESI probe. The mobile phases were (A) 60:40 acetonitrile or methanol/water with 10 mM ammonium formate and 0.1% formic acid and (B) 90:10:0.1 isopropanol/acetonitrile or methanol/water with the same type of mobile phase modifiers.

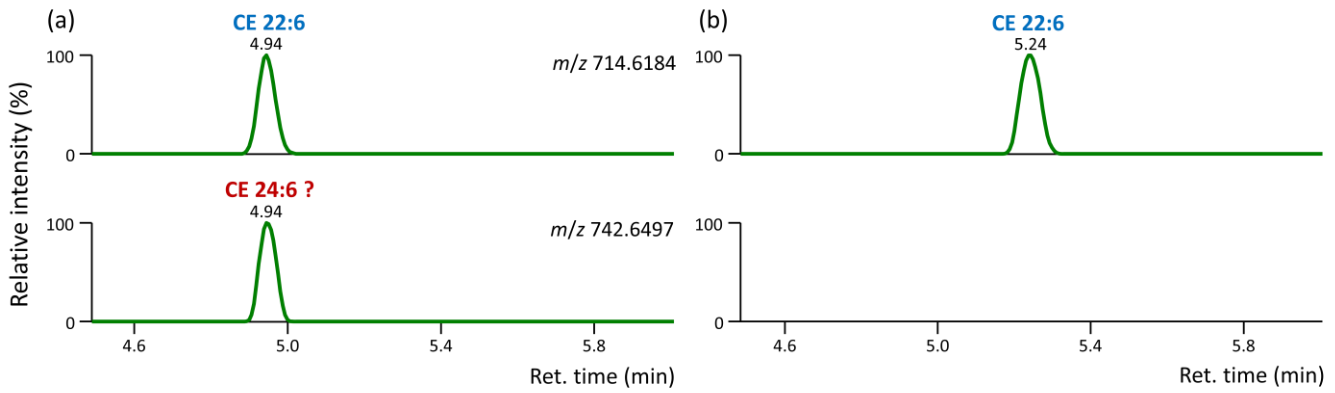

Figure S9. LC-MS lipidomic profiling of CE species in human plasma extracts (NIST SRM 1950). (**a**,**b**) Extracted ion chromatograms corresponding to CE 22:6 (*m*/*z* 714.6184) and CE 24:6 (*m*/*z* 742.6497), indicating true lipids (in blue) and misidentified lipids (in red) based on MS1 accurate mass only. (**a**) Data acquired with mobile phases containing acetonitrile and including the grounding union connecting the LC column and ESI probe; (**b**) data acquired with mobile phases containing methanol instead of acetonitrile and including the grounding union connecting the LC column and ESI probe. The mobile phases were (A) 60:40 acetonitrile or methanol/water with 10 mM ammonium formate and 0.1% formic acid and (B) 90:10:0.1 isopropanol/acetonitrile or methanol/water with the same type of mobile phase modifiers.

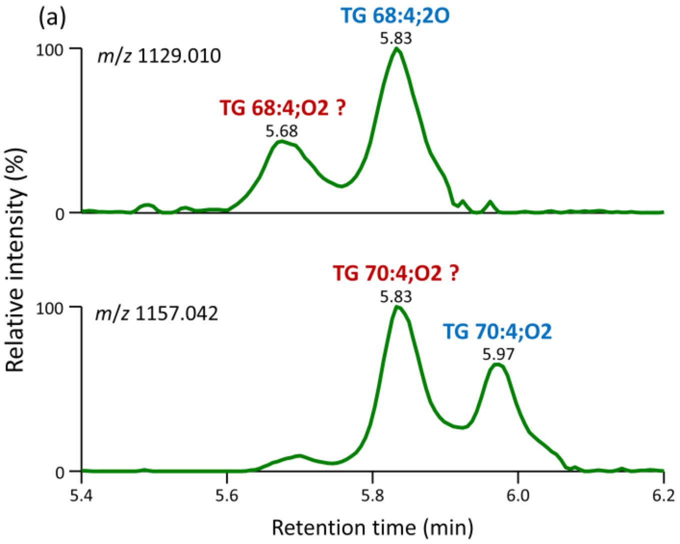

Figure S10. LC-MS lipidomic profiling of TG-EST species in mouse WAT extracts. (**a**,**b**) Extracted ion chromatograms corresponding to TG 68:4;2O (*m*/*z* 1129.010) and TG 70:4;O2 (*m*/*z* 1157.042), indicating true lipids (in blue) and misidentified lipids (in red) based on MS1 accurate mass only. (**a**) Data acquired with mobile phases containing acetonitrile and including the grounding union connecting the LC column and ESI probe; (**b**) data acquired with mobile phases containing methanol instead of acetonitrile and including the grounding union connecting the LC column and ESI probe. The mobile phases were (A) 60:40 acetonitrile or methanol/water with 10 mM ammonium formate and 0.1% formic acid and (B) 90:10:0.1 isopropanol/acetonitrile or methanol/water with the same type of mobile phase modifiers.

Figure S11. Examples of MS/MS spectra of true and misidentified TG species in 3T3-L1 cell extracts. (**a**) True lipid TG 52:2, (**b**) misidentified lipid TG 52:2, (**c**) true lipid TG 52:3 (adjacent lipid to misidentified lipid TG 52:2). Upper panels: acquired MS/MS spectra; bottom panels: in-silico MS/MS spectra from MS-DIAL. Acquired precursor ions (accurate mass) and theoretical precursor ions (exact mass) from MS-DIAL are also provided.

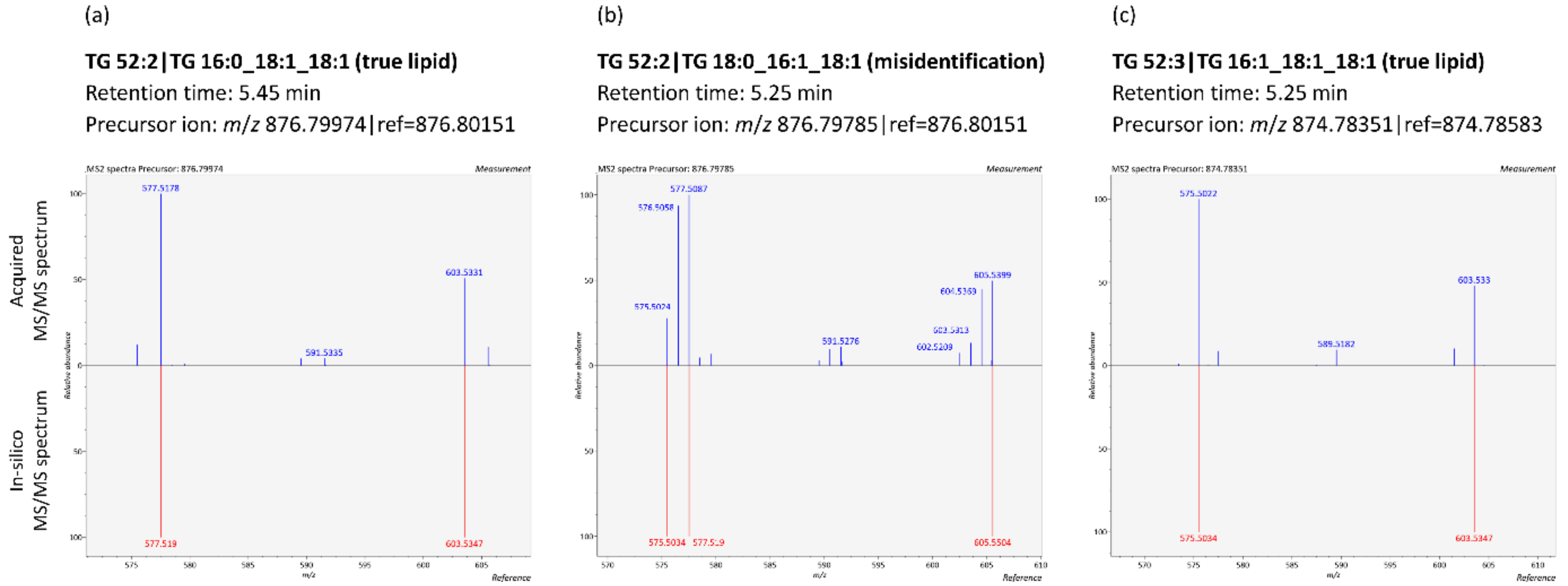

Figure S12. Examples of MS/MS spectra of true and misidentified TG species in 3T3-L1 cell extracts. (**a**) True lipid TG 54:2, (**b**) misidentified lipid TG 54:2, (**c**) true lipid TG 54:3 (adjacent lipid to misidentified lipid TG 54:2). Upper panels: acquired MS/MS spectra; bottom panels: in-silico MS/MS spectra from MS-DIAL. Acquired precursor ions (accurate mass) and theoretical precursor ions (exact mass) from MS-DIAL are also provided.

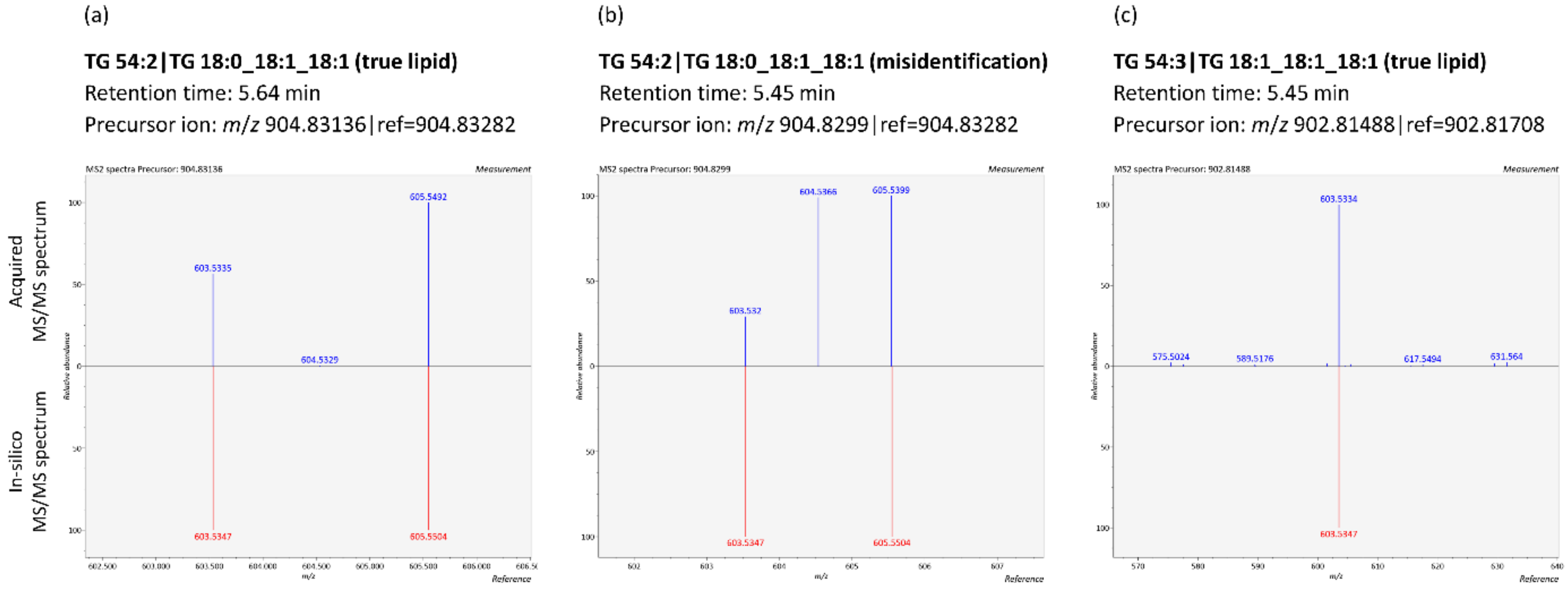

Figure S13. Examples of MS/MS spectra of true and misidentified TG species in human serum extracts. (**a**) True lipid TG 52:3, (**b**) misidentified lipid TG 52:3, (**c**) true lipid TG 52:4 (adjacent lipid to misidentified lipid TG 52:3). Upper panels: acquired MS/MS spectra; bottom panels: in-silico MS/MS spectra from MS-DIAL. Acquired precursor ions (accurate mass) and theoretical precursor ions (exact mass) from MS-DIAL are also provided.

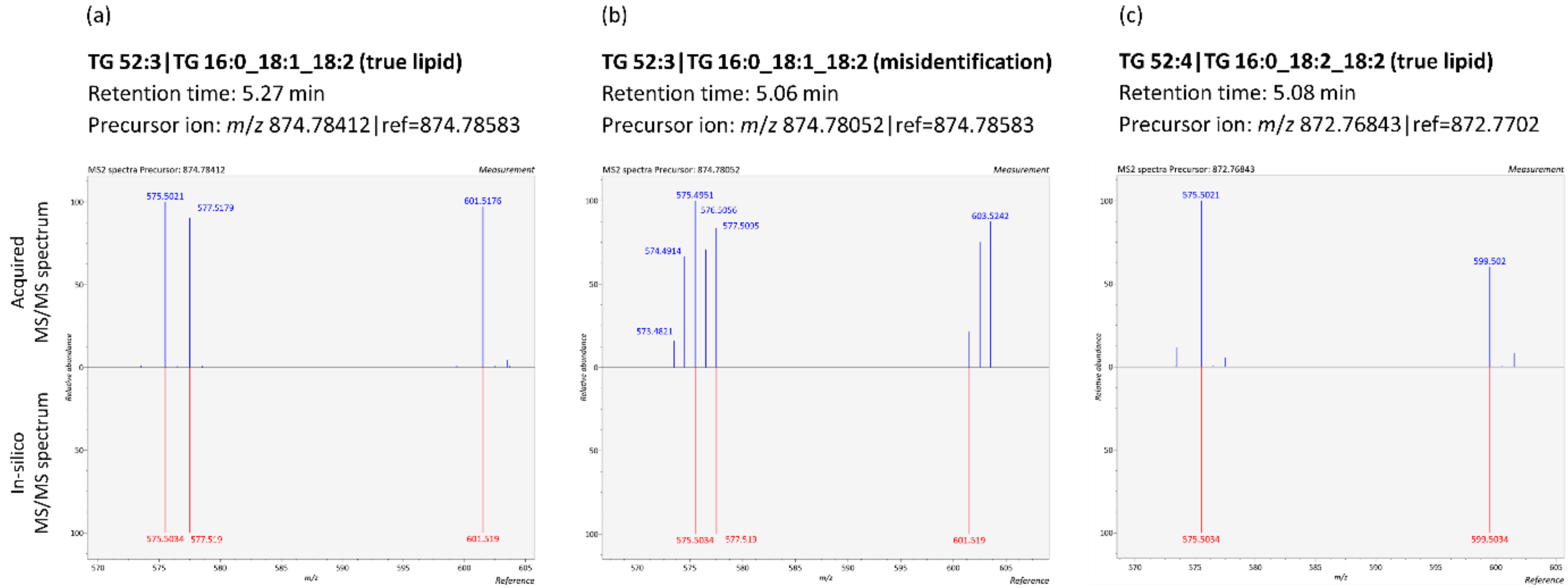

Figure S14. Examples of MS/MS spectra of true and misidentified TG species in mouse WAT extracts. (**a**) True lipid TG 54:2, (**b**) misidentified lipid TG 54:2, (**c**) true lipid TG 54:3 (adjacent lipid to misidentified lipid TG 54:2). Upper panels: acquired MS/MS spectra; bottom panels: in-silico MS/MS spectra from MS-DIAL. Acquired precursor ions (accurate mass) and theoretical precursor ions (exact mass) from MS-DIAL are also provided.

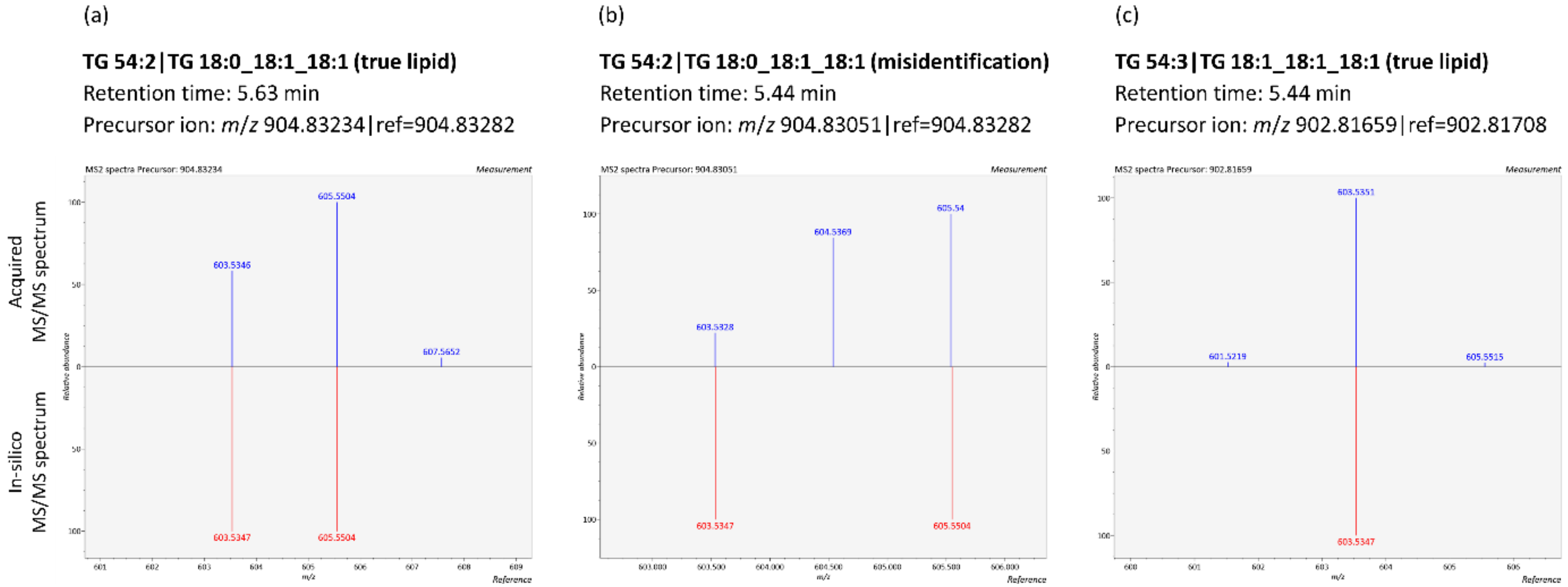

Supplement: Supplementary file 1 [file metabolites-13-00966-s001.zip › metabolites-2568263-Supplementary_Materials_Figures S1-S14.pdf]
